# Ranking candidate genes in rat models of type 2 diabetes

**DOI:** 10.1186/1742-4682-6-12

**Published:** 2009-07-03

**Authors:** Lars Andersson, Greta Petersen, Fredrik Ståhl

**Affiliations:** 1 Department of Cell and Molecular Biology-Genetics, Göteborg University, Box 462, SE 40530 Göteborg, Sweden; 2 School of Health Science, University Collage of Borås, SE-501 90 Borås, Sweden

## Abstract

**Background:**

Rat models are frequently used to find genomic regions that contribute to complex diseases, so called quantitative trait loci (QTLs). In general, the genomic regions found to be associated with a quantitative trait are rather large, covering hundreds of genes. To help selecting appropriate candidate genes from QTLs associated with type 2 diabetes models in rat, we have developed a web tool called Candidate Gene Capture (CGC), specifically adopted for this disorder.

**Methods:**

CGC combines diabetes-related genomic regions in rat with rat/human homology data, textual descriptions of gene effects and an array of 789 keywords. Each keyword is assigned values that reflect its co-occurrence with 24 different reference terms describing sub-phenotypes of type 2 diabetes (for example "insulin resistance"). The genes are then ranked based on the occurrences of keywords in the describing texts.

**Results:**

CGC includes QTLs from type 2 diabetes models in rat. When comparing gene rankings from CGC based on one sub-phenotype, with manual gene ratings for four QTLs, very similar results were obtained. In total, 24 different sub-phenotypes are available as reference terms in the application and based on differences in gene ranking, they fall into separate clusters.

**Conclusion:**

The very good agreement between the CGC gene ranking and the manual rating confirms that CGC is as a reliable tool for interpreting textual information. This, together with the possibility to select many different sub-phenotypes, makes CGC a versatile tool for finding candidate genes. CGC is publicly available at .

## Background

Type 2 diabetes is one of the fastest growing health problems all over the world and accounts for more than 90% of all cases of diabetes. The total number of people with diabetes worldwide was estimated to be between 151 and 171 million in 2000, and is expected to rise to 366 million by the year of 2030 [1]. The disease is defined by chronically elevated plasma glucose levels, but the development of the disorder is complex, depending on both environmental as well as multiple genetic factors. This complexity seriously complicates the study of the disease. Here, animal models are very useful since their environment can be well controlled and inbred animals ensure a homogenous genetic background [2]. Consequently, inbred rat strains predisposed for developing phenotypes closely resembling type 2 diabetes have frequently been used to explore the relation between the diabetes phenotype and the genotype.

In most genetic studies of type 2 diabetes using rat models, two different inbred strains have been utilised, Goto-Kakazaki (GK) and Otsuka Long-Evans Tokushima fatty (OLETF). Rats from both these strains spontaneously develop phenotypes that resemble human type 2 diabetes. The GK-rat is a non-obese model of type 2 diabetes that is characterised by glucose intolerance, insulin resistance, hyperinsulinaemia, altered insulin secretion and reduced beta cell mass [3,4]. The OLETF-rat on the other hand is an obese model of type 2 diabetes. At the age of 25 weeks, male OLETF-rats develop a diabetic syndrome in nearly 100% of the cases [5]. OLETF-rats lack the cholecystokinin-1 receptor, which has been shown to lead to increased food intake due to decreased satiety [6]. The obesity in these rats is secondary to increased food intake and exercise is effective at preventing diabetes in OLETF-rats [6,7].

DNA-marker characterizations of offspring from back- and F2-crosses of inbred non-diabetic and diabetic rat strains (i.e. most often GK or OLETF) reveal regions associated with the trait under study, so called QTL (Quantitative Trait Locus) analysis [8,9]. In most studies, traits quantified in the type 2 diabetes models include glucose level, insulin level, body weight, gland mass, lipid level or body fat amount. At present, at least 70 Niddm-(non insulin dependent diabetes mellitus) QTLs have been reported in rat [10]. However, limitations in the number of animals used to define a given QTL most often result in very large suggestive genomic regions covering several hundred genes. This poses a great problem in further search for the disease-causing gene(s) and thus a limitation in the number of potential candidate genes is of great value.

In order to facilitate the search for such candidate genes, we have previously developed a web-tool that uses textual gene information as a basis for gene ranking. This tool was adopted for arthritis phenotypes and proved to be very successful in ranking appropriate candidate genes [11]. Based on these experiences, we are now releasing a similar tool for the diabetes rat model. However, the larger number of QTL-regions and the multitude of phenotypic measurements used in the diabetes rat models have raised the need for a much more extended web-tool with new functions for handling the more complex features. In this paper we present this new tool together with an evaluation of its functions.

## Methods

Previously, we have developed a web-based tool that facilitates the identification of candidate genes that contribute to experimentally induced autoimmune arthritis. This application, called Candidate Gene Capture (CGC), was created by combining QTL regions in rat with human gene homology data, descriptions of phenotypic gene effects and selected keywords using the word "arthritis" as a unifying selection criterion [11]. Now, we are building a related web-tool using QTL-regions from diabetic rat models, a large set of diabetes-relevant keywords and a range of different selection criteria.

### QTL data

QTL information containing QTL-symbols, descriptions and flanking markers were collected from the rat genome databases Ratmap  and RGD . This information was stored in a MySQL-table called "QTL". The handling of the data was done according to the same protocol as for the CGC arthritis web-tool [11].

### Gene homology data

Gene homology data between rat and human was assembled as previously described [11]. In addition, the human genomic regions homologous to each rat QTL are now automatically loaded, based on flanking markers and homology data. This enables an easy updating of databases containing gene homology data between rat and human.

### Downloading Gene Functional Data

The OMIM (Online Mendelian Inheritance in Man) database  contains a comprehensive record of gene function and clinical data, which is used as a source for keyword querying in the CGC application. For each human gene, gene function information is downloaded from OMIM and stored in a table labelled "OMIMdata".

### Selecting reference terms and ranking keywords

A reference term is the selection criterion used to estimate the association of a given keyword to a phenotype of interest. In total, 24 reference terms related to different aspects of metabolic disorders were selected from the literature. Keywords were selected from MeSH terms as well as other terms associated with metabolic disorders.

For each keyword, a so called relevance index was calculated by dividing the number of PubMed  abstracts containing both the keyword and the selected reference term with the number of PubMed abstracts containing the keyword alone. The ratio is multiplied by 100 to get the percentage figures. In total, 789 keywords are used in the application.

Keywords with relevance indices of less than 0.1 are omitted since they will have very little impact on the gene ranking. Depending on which reference term that is being used, the list of available keywords varies widely. For the reference term "diabetes", 330 of the keywords are found to be relevant and included in the search, whereas for the reference term "diabetic foot" only 24 relevant keywords are found.

Furthermore, a subset of 28 keywords was selected based on how often they occur in literature on diabetic disorders. This subset of keywords was used in a quick version of the CGC diabetes application. When ranking genes with high CGC scores, keywords with low keyword values have minor impact on the ranking. By excluding these keywords from the analysis, the quick version of the application will run much faster with low risk of missing highly ranked genes. The keywords were stored in MySQL-tables called "DiabetesKeywords" and "DiabetesKeywordsShort".

All reference terms and keywords included in the CGC application are available in Additional file 1.

### Web application

QTL data from the MySQL-table "QTL" has been made accessible through an introductory web page . Here, the user can find a QTL of interest by searching for a QTL-symbol, a brief functional description or a chromosomal position. When a QTL has been selected, the individual QTL is presented together with a list of known orthologous rat genes and human genes within the homologous interval.

**Figure 1:**
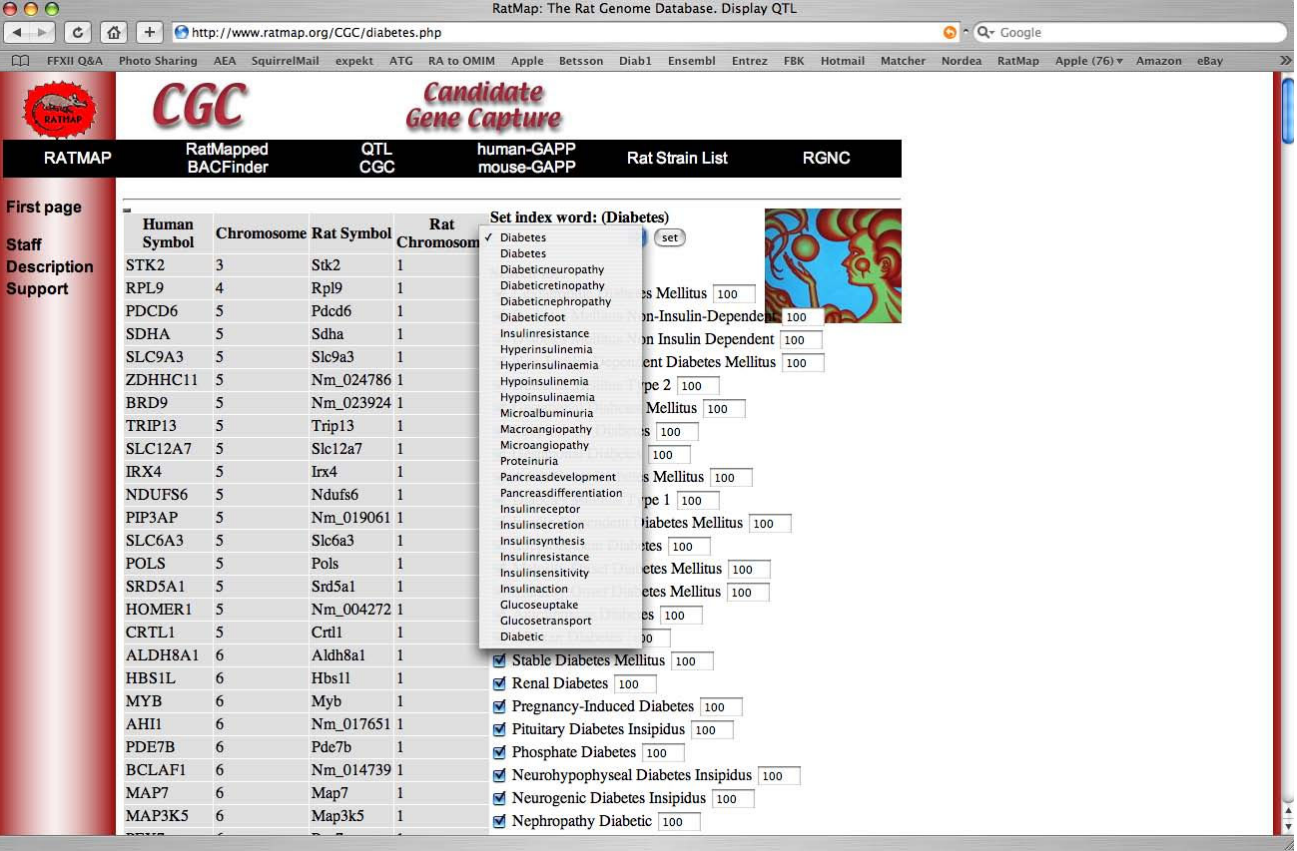
**Snapshot of the CGC Diabetes application**. The CGC-Diabetes application involves the selection of reference terms to which the keywords are to be compared.

To search this gene list for the most likely candidate genes, the user first selects a reference term reflecting a sub-phenotype of interest (i.e. glucose tolerance, insulin resistance etc). A list of keywords with relevant keyword indices above 0.1 is generated. The user may select or deselect an optional number of keywords, and/or change relevance indices. The user may also assign up to ten keywords of his/her own choice and the relevance index for each new keyword is calculated (Figure 1).

When performing the query, the OMIM-text for each of the homologous human genes is scanned for all keywords selected. The keyword indices of all keywords found within the OMIM-text of each gene are added to a total score. A list of all matching genes is presented ranked by their total score.

### Manual evaluation

In order to evaluate the CGC tool we manually rated genes found within four randomly chosen QTLs (*Niddm8*, *Niddm18*, *Niddm38 *and *Niddm46*) [12-15]. The genes were rated from 1 to 5, 1 meaning that the connection to diabetes was obvious and 5 meaning that we found no connection to diabetes whatsoever. Our manual rating was then compared with the ranking obtained from the CGC tool using "diabetes" as reference term. In two of the evaluated QTLs, a large number of genes with at least one matching keyword were found (*Niddm18*; 72 genes, *Niddm46*; 80 genes). The other two QTLs resulted in a lower number of matching genes (*Niddm8*; 9 genes, *Niddm38*; 16 genes). In the two smaller QTLs, all genes with at least one matching keyword were manually rated, whereas in the two larger ones, only genes with a CGC score of 15 and above were manually rated. The manual ratings of the genes were done without prior knowledge of their CGC-scores.

## Results

To evaluate the CGC application, we made a manual rating of genes within four randomly chosen QTLs (*Niddm8*, *Niddm18*, *Niddm38 *and *Niddm46*). Genes within each QTL were divided into five categories according to how likely they were to infer susceptibility to type 2 diabetes: 1 – "Obvious" candidate gene, 2 – "Likely" candidate gene, 3 – "Possible" candidate gene, 4 – "Unlikely" candidate gene and 5 – "Irrelevant" gene. The outcome of the manual evaluation was then compared to a ranking made by the CGC application. This CGC ranking was made with "diabetes" as the reference term. (Note that the database is updated on a regular basis, hence the present version of CGC may not coincide totally with this manual evaluation.) Detailed descriptions of the top ranked genes in each QTL are available as Additional file 2.

### Niddm8

In total, 9 genes were ranked by the CGC application. *SHC1 *and *ENSA *were ranked as the two top candidates with CGC points exceeding 100. No "obvious" candidate gene was found in the manual inspection, but *SHC1 *and *ENSA *were considered to be the only two "likely" candidate genes. The remaining seven genes were all considered to be "irrelevant" in the manual rating. The mean CGC point in this group of genes was 7.5, ranging from 2.4 to 20.3.

### Niddm18

In total, 72 genes were ranked by the CGC application. In the manual inspection only genes with a CGC ranking of 15 and above were evaluated. *GCK *was ranked as the outstanding top candidate and was also considered to be an "obvious" candidate gene in the manual inspection. Two additional genes were rated as "obvious" candidate genes in the manual rating: *GC *and *NKX6A*. These two genes were ranked as number 2 and 5 in the CGC ranking. Two genes ranked 3 and 4 (*CCKAR *and *WFS1*) were both manually rated as "likely" candidate genes.

A middle group of 18 genes had a mean manual ranking of 3.9 and ranged from 2 to 5. Specifically, three genes were manually ranked 2; *CD38*, *SLC2A9 *and *SLC5A1*. The mean CGC point in this middle group was 22.4, ranging from 15 to 75.5. The remaining 49 ranked genes had a mean CGC score of 4.7 and were not manually evaluated.

### Niddm38

In total, 16 genes were ranked by the CGC application. Five genes (*RRAD*, *FANCA*, *CETP*, *FOXC2 *and *HP*) obtained a CGC score above 100. The *RRAD *and *FOXC2 *genes were manually rated as "obvious" candidate genes and the remaining three genes were rated as "likely".

A middle group of 7 genes had a mean manual ranking of 3.0 and ranged from 2 to 5. Specifically, three genes were manually ranked 2; *AGRP*, *CDH13 *and *HSD11B2*. The mean CGC point in this middle group was 44.4 ranging from 18.7 to 88.8. The remaining 4 ranked genes had a mean CGC score of 12.8 and were all manually rated as 5.

### Niddm46

In total, 80 genes were ranked by the CGC application. In the manual inspection, only genes with a CGC ranking of 15 and above were evaluated. Nine genes (*GAD1*, *NEUROD1*, *DPP4*, *MAPK8IP1*, *GCG*, *GPD2*, *CD59*, *CAT*, *FUT7*) obtained a CGC score above 100. Five of these genes (*NEUROD1*, *DPP4*, *MAPK8IP1*, *GCG*, *GPD2*) were manually rated as "obvious candidate genes". These genes were ranked among the 6 best candidate genes by the CGC application.

A middle group of 15 genes had a mean manual ranking of 3.5 and ranged from 2 to 5. Specifically, two genes were manually rated 2; *RXRA *and *SLC2A8*. The mean CGC point in this group was 29.7 ranging from 15.5 to 66.2. The remaining 56 ranked genes had a mean CGC score of 2.3 and were not manually evaluated.

#### Evaluating the significance of different reference terms

To evaluate how much the results from CGC differ when using different reference terms, for one single QTL (*Niddm46*) we calculated the difference in ranking position between the results obtained from searches using all reference terms. For example, the gene NEUROD1 is ranked 1 when using "diabetes" as the reference term, but ranked 6 when using "glucose uptake" as a reference term. Hence, the difference in ranking position is 6-1 = 5. The sum of such differences between two reference terms was used as an estimate of similarity in gene ranking between two reference terms. This calculation was made for the ten genes ranked highest by CGC in Niddm46 for all reference terms and all these gene rankings were compared with each other.

To get an overview of which reference terms that result in the most similar rankings, the sum differences between all reference terms were used to construct a tree.

**Figure 2:**
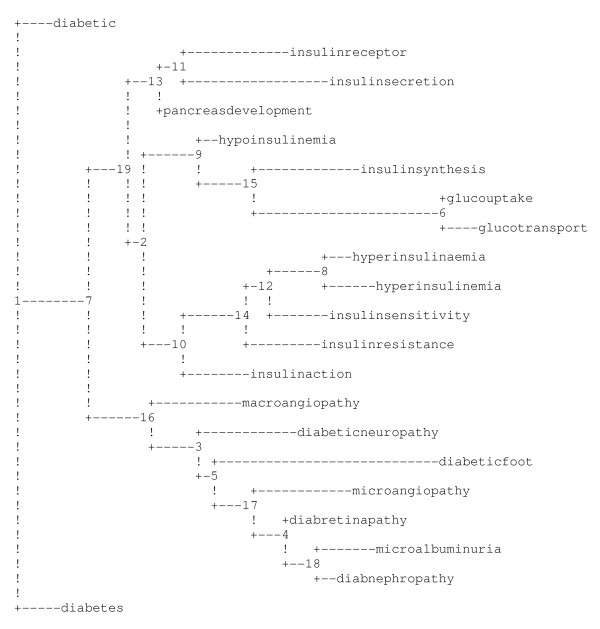
**Comparison of results using different reference terms**. The horizontal branches of the tree illustrate the distances between reference terms. Two reference terms with a short distance separating them will rank genes in a similar way, while terms with larger distances between them will generate rankings where the order of genes will be more different.

The tree was constructed using the program "FITCH" from Phylip (Phylogeny interference package version 3.66) [16]. FITCH was developed to create phylogenic trees based on distances computed from molecular sequences, restriction sites or fragment distances or from genetic distances computed from gene frequencies. FITCH is based on the Fitch-Margoliash method, a distance based optimization, which searches for a tree with the smallest squared distance between the computed distances and their predictions from the tree. FITCH estimates phylogenies from distance matrix data under the "additive tree model" according to which the distances are expected to equal the sums of branch lengths between the species compared. For our tree however, we used the differences in ranking positions of the CGC as the distance matrix (Figure 2). Four reference terms were omitted from this final presentation because of limitations in the number of ranked genes.

The rankings used to construct this tree were based on a quick version of the CGC application. In this quick version only 28 keywords are used in each query. The 28 keywords were manually selected based on their frequency in diabetes related literature as well as on high keyword values. This quick version is available through the website.

## Discussion

A recurrent problem when performing genetic studies of complex diseases, such as type 2 diabetes, is that genomic regions found to be associated with the phenotype are rather large. Finding appropriate candidates within these regions is generally not a simple task. In this paper we present a tool (CGC) that facilitates the search for candidate genes within type 2 diabetes associated QTL regions in rat. This is done by analysing textual gene information for a large set of keywords weighted against a set of phenotypical reference terms. The outcome of the analysis is a ranking of all genes in a selected QTL region.

### Niddm8

The two genes that obtained more than 100 CGC points in *Niddm8 *were also manually considered to be the best candidate genes (manual rating 2). The seven remaining genes all obtained less than 30 CGC points and were also manually considered to be "irrelevant".

### Niddm18

Out of the five genes that obtained more than 100 CGC points in *Niddm18*, four were manually considered to be "obvious" candidate genes and the fifth was rated as "likely ". The 18 remaining genes with CGC points between 15 and 75.5 were all manually rated as "unlikely" or "irrelevant" except for one that was rated as "possible" and three that were rated as "likely" candidate genes; *CD38*, *SLC2A9 *and *SLC5A1*.

Although rather briefly mentioned in OMIM, CD38 participates in the Ca-dependent activation of insulin secretion [17]. Autoantibodies against CD38 in several type 2 diabetes patients also suggest an important role in the disease, however these results are under debate [18]. *CD38 *is not reaching 100 CGC points which most likely is due to lack of the word "diabetes" in OMIM. Still, CGC rates *CD38 *quite high (49.6 points) because of hits from four separate keywords.

SLC2A9 and SLC5A1 are both glucose transporters over the cell membrane [19,20] and are as such interesting candidate genes for diabetes. However, neither SLC2A9 nor SLC5A1 has been shown to be closely associated with diabetes and this is reflected in the descriptive text in OMIM, which is very brief. Thus, the difference between CGC and our manual rating for these two glucose transporters is not based so much on evidence as on human expectations.

### Niddm38

Out of the five genes that obtained more than 100 CGC points in *Niddm38*, two were manually considered to be "obvious" candidate genes and three were rated as "likely". Among the eleven remaining genes eight were manually rated as "possible", "unlikely" and "irrelevant", whereas three were rated as "likely"; *AGRP*, *CDH13 *and *HSD11B2*.

AGRP normally regulates body weight in mice through central melanocortin receptors [21]. AGRP is increased in obese men and AGRP levels are correlated with various parameters of obesity [22]. Although *AGRP *does not reach 100 CGC points, it still obtains a high score (88.8 points), placing it at the fifth position among the candidates within this QTL.

CDH13 is expressed in endothelial and smooth muscle cells, where it is positioned to interact with adiponectin. CDH13 is a glycosylphosphatidylinositol-anchored extracellular protein, and may act as a coreceptor for the transmission of adiponectin metabolic signals [23]. Since adiponectin is a hormone secreted by adipocytes that regulate energy, glucose and lipid metabolism, *CDH13 *is rated high in our evaluation. Furthermore, several studies of human population suggest an increased risk of type 2 diabetes as a consequence of low adiponectin levels [24]. In the CGC application, *CDH13 *obtains 38.9 points from only one single matching keyword ("adiponectin"). However, the close connection between adiponectin and type 2 diabetes is not discussed further in the OMIM-text explaining the low CGC point.

HSD11B2 confers specificity to the mineralocorticoid receptor (MR) by converting biologically active glucocorticoids (cortisol) to inactive metabolites (cortison). We find the gene interesting in the manual evaluation since elevated cortisol levels contribute to the development of the entire spectrum of the metabolic syndrome, including visceral obesity, insulin resistance and dyslipidemia [25,26]. In CGC, *HSD11B2 *is ranked tenth, obtaining 35.1 CGC points due to as much as nine matching keywords, although each contributes with a relatively small amount.

### Niddm46

Out of the nine genes that obtained more than 100 CGC points in *Niddm46*, five were manually considered to be "obvious" candidate genes, two were rated as "likely", and another two were rated as "possible". The 15 remaining genes with CGC points between 15 and 66,2 were all manually rated as "possible" or "unlikely" except for one that was rated as "irrelevant" and two genes that were rated as "likely"; *RXRA*, and *SLC2A8*.

RXRA is a versatile regulator of metabolic function including glucose and lipid homeostasis. RXRA is a member of the Retinoid × Receptor family which is reported to play an important role in different metabolic disorders including type 2 diabetes [27]. Due to its multiple functions, the glucose regulating function of RXRA is only briefly mentioned in OMIM resulting in a CGC point of 32.3.

SLC2A8 is another glucose transporter and the difference in rating between CGC and our manual evaluation is explained by the same argument as stated for *SLC29A2 *and *SLC5A1 *above.

*CD59 *has 121.9 CGC points but is only considered to be a "possible" candidate gene in our evaluation. The reason for this discrepancy is that although *CD59 *is very much involved in the diabetes phenotype, it seems to be responsible for the vascular changes that follow from type 2 diabetes. Thus, several keywords fit very well, but the CGC application cannot distinguish a secondary function from a primary.

FUT7 has 105.6 CGC points but is only considered to be a "possible" candidate gene in our evaluation. The reason for this discrepancy is that the OMIM text makes a rather extensive description of one patient that has a homozygous loss of function mutation in *FUT7*. One of the symptoms mentioned was noninsulin-dependent diabetes, which brings 100 points to the gene although it is stated that the connection is unclear.

In summary, for all four QTLs, a total of 21 genes obtained a CGC score exceeding 100. Of these genes, 11 were manually rated as "obvious" candidate genes, 8 were rated as "likely" candidate genes and 2 were rated as "possible" candidate genes.

In the QTLs *Niddm8 *and *Niddm38*, all genes with a CGC score less than 100 were manually evaluated. In *Niddm18 *and *Niddm46*, only genes with a score of 15 to 100 were manually evaluated. Out of these genes, 8 were considered to be "likely" candidate genes, 7 were considered to be "possible" candidate genes, 17 were considered to be "unlikely" candidate genes and 18 were considered to be "irrelevant". Thus, no genes with a CGC score less than 100 were considered to be an "obvious" candidate gene.

Overall, this comparison between our manual evaluation and the CGC ranking shows an exceptionally good agreement. The manual consideration did not only involve reading the OMIM text but was also based on exploration of a great number of additional references and took considerable time to undertake. This is in contrast to the much faster process of simply running the CGC application.

#### Using different reference terms

As shown above, using "diabetes" as the reference term works very well when searching for genes related to the disease. The term diabetes is rather general though, and many phenotypes are categorised under this diagnosis. If the trait under study is well specified, a more specific reference term will probably be more informative. The physiologic phenotypes of the different inbred rats used to construct the Niddm-QTLs are well studied and the resulting candidate genes will probably be more accurate if the choice of reference term reflects these phenotypes. For example, if the GK-rat was used, reference terms like "glucose intolerance", "insulin resistance" and "hyperinsulinaemia" would probably be good choices, since these are all among the defined characteristics of this strain. Another thing to bear in mind is that each diabetes-QTL analysis is constructed by quantifying a specific trait. These traits include "glucose level", "insulin level", "body weight", "gland mass", "lipid level" and "body fat amount". Selecting reference terms corresponding to the quantified trait is thus probably a good idea.

#### Comparison of results when using different reference terms

To evaluate the use of different reference terms, we compared the rankings for all 24 reference terms within one single QTL (*Niddm46*). By calculating the sum of differences in gene position obtained with different reference terms, we could measure the similarity in rankings. Sums of differences in gene rankings were calculated for all pair wise comparisons between reference terms. These sums were used as a distance matrix for constructing a "phylogenetic" tree using the FITCH software [16]. The tree makes it possible to get an overview of how similar the reference terms are in ranking possible candidate genes.

In the tree, certain reference terms are grouped together. For example, there is a group of five reference terms that are all associated with insulin ("insulin action", "insulin resistance", "insulin sensitivity", "hyperinsulinemia" and "hyperinsulinaemia"). Other reference terms that cluster together are "glucose uptake" and "glucose transport" as well as "microalbuminurea" and "diabetic nephropathy". In all, the distances between and clustering of reference terms in the tree are very close to what can be expected from a functional perspective. Thus, these results clearly demonstrate that functionally related terms generate, more or less, the same candidate genes. Consequently, the tree can be useful as guidance for choosing reference terms.

Since the ranking of genes is based on matching keywords and their reference points, the distances between reference terms in the tree do not only reflect gene ranking, but also the order in which the keywords are ranked. Based on our analysis it seems that the total point for each gene in searches with two closely related reference terms may vary widely, but the order of the gene ranking will still be very similar. The same goes for the keywords included in the search and is merely a reflection of the frequency of the reference terms among PubMed abstracts. This is most likely caused by the tendency of certain keywords to co-occur at a higher frequency, whereas more specific reference terms will be mentioned in fewer papers and hence generate lower points. However, the order of the keywords will be more or less the same using related reference terms.

## Conclusion

We believe that the very good agreement between our manual rating for the four evaluated QTLs (*Niddm8*, *Niddm18*, *Niddm38 *and *Niddm46*) and the ranking made by the CGC application proves that the application makes reliable predictions when selecting candidate genes for diabetes. Furthermore, the differences in gene ranking observed when using different reference terms (visualised in Figure 1) indicate that the application will generate candidate genes appropriate for each sub-phenotype. Overall, we believe that the CGC application can be of great use when selecting candidate genes for phenotypes related to type 2 diabetes within defined QTL regions.

## Competing interests

The authors declare that they have no competing interests.

## Authors' contributions

LA carried out the programming of the CGC application, contributed with original ideas and drafted the manuscript. GP created the rat/human comparative database and implemented it in the CGC application. FS supervised the project, contributed with original ideas and took part in the preparation of the manuscript. All authors read and approved the final manuscript.

## Supplementary Material

Additional file 1 **References and keywords.** Click here for file

Additional file 2 **Detailed description of high-ranked genes within the four investigated QTLs.** Click here for file

## References

[B1] Kasuga M (2006). Insulin resistance and pancreatic beta cell failure. J Clin Invest.

[B2] Srinivasan K, Ramarao P (2007). Animal models in type 2 diabetes research: an overview. Indian J Med Res.

[B3] Portha B (2005). Programmed disorders of beta-cell development and function as one cause for type 2 diabetes? The GK rat paradigm. Diabetes Metab Res Rev.

[B4] Cox RD, Brown SD (2003). Rodent models of genetic disease. Curr Opin Genet Dev.

[B5] Kawano K, Mori S, Hirashima T, Man ZW, Natori T (1999). Examination of the pathogenesis of diabetic nephropathy in OLETF rats. J Vet Med Sci.

[B6] Moran TH, Bi S (2006). Hyperphagia and obesity in OLETF rats lacking CCK-1 receptors. Philos Trans R Soc Lond B Biol Sci.

[B7] Shima K, Shi K, Mizuno A, Sano T, Ishida K, Noma Y (1996). Exercise training has a long-lasting effect on prevention of non-insulin-dependent diabetes mellitus in Otsuka-Long-Evans-Tokushima Fatty rats. Metabolism.

[B8] Lander ES, Schork NJ (1994). Genetic dissection of complex traits. Science.

[B9] Ktorza A, Bernard C, Parent V, Penicaud L, Froguel P, Lathrop M, Gauguier D (1997). Are animal models of diabetes relevant to the study of the genetics of non-insulin-dependent diabetes in humans?. Diabetes Metab.

[B10] The Rat Genome Database, RGD. http://www.rgd.mcw.edu/.

[B11] Andersson L, Petersen G, Johnson P, Stahl F (2005). A web tool for finding gene candidates associated with experimentally induced arthritis in the rat. Arthritis Res Ther.

[B12] Gauguier D, Froguel P, Parent V, Bernard C, Bihoreau MT, Portha B, James MR, Penicaud L, Lathrop M, Ktorza A (1996). Chromosomal mapping of genetic loci associated with non-insulin dependent diabetes in the GK rat. Nat Genet.

[B13] Moralejo DH, Ogino T, Zhu M, Toide K, Wei S, Wei K, Yamada T, Mizuno A, Matsumoto K, Shima K (1998). A major quantitative trait locus co-localizing with cholecystokinin type A receptor gene influences poor pancreatic proliferation in a spontaneously diabetogenic rat. Mamm Genome.

[B14] Ogino T, Moralejo DH, Zhu M, Toide K, Wei S, Wei K, Yamada T, Mizuno A, Matsumoto K, Shima K (1999). Identification of possible quantitative trait loci responsible for hyperglycaemia after 70% pancreatectomy using a spontaneously diabetogenic rat. Genet Res.

[B15] Watanabe TK, Okuno S, Oga K, Mizoguchi-Miyakita A, Tsuji A, Yamasaki Y, Hishigaki H, Kanemoto N, Takagi T, Takahashi E (1999). Genetic dissection of "OLETF," a rat model for non-insulin-dependent diabetes mellitus: quantitative trait locus analysis of (OLETF × BN) × OLETF. Genomics.

[B16] Felsenstein J (2005). PHYLIP (Phylogeny Inference Package) version 3.6. Distributed by the author.

[B17] Johnson JD, Misler S (2002). Nicotinic acid-adenine dinucleotide phosphate-sensitive calcium stores initiate insulin signaling in human beta cells. Proc Natl Acad Sci USA.

[B18] Ikehata F, Satoh J, Nata K, Tohgo A, Nakazawa T, Kato I, Kobayashi S, Akiyama T, Takasawa S, Toyota T, Okamoto H (1998). Autoantibodies against CD38 (ADP-ribosyl cyclase/cyclic ADP-ribose hydrolase) that impair glucose-induced insulin secretion in noninsulin- dependent diabetes patients. J Clin Invest.

[B19] Wright EM, Loo DD, Panayotova-Heiermann M, Lostao MP, Hirayama BH, Mackenzie B, Boorer K, Zampighi G (1994). 'Active' sugar transport in eukaryotes. J Exp Biol.

[B20] Phay JE, Hussain HB, Moley JF (2000). Cloning and expression analysis of a novel member of the facilitative glucose transporter family, SLC2A9 (GLUT9). Genomics.

[B21] Ollmann MM, Wilson BD, Yang YK, Kerns JA, Chen Y, Gantz I, Barsh GS (1997). Antagonism of central melanocortin receptors in vitro and in vivo by agouti-related protein. Science.

[B22] Katsuki A, Sumida Y, Gabazza EC, Murashima S, Tanaka T, Furuta M, Araki-Sasaki R, Hori Y, Nakatani K, Yano Y, Adachi Y (2001). Plasma levels of agouti-related protein are increased in obese men. J Clin Endocrinol Metab.

[B23] Hug C, Wang J, Ahmad NS, Bogan JS, Tsao TS, Lodish HF (2004). T-cadherin is a receptor for hexameric and high-molecular-weight forms of Acrp30/adiponectin. Proc Natl Acad Sci USA.

[B24] Kadowaki T, Yamauchi T, Kubota N, Hara K, Ueki K, Tobe K (2006). Adiponectin and adiponectin receptors in insulin resistance, diabetes, and the metabolic syndrome. J Clin Invest.

[B25] Bjorntorp P (1993). Visceral obesity: a "civilization syndrome". Obes Res.

[B26] Oltmanns KM, Dodt B, Schultes B, Raspe HH, Schweiger U, Born J, Fehm HL, Peters A (2006). Cortisol correlates with metabolic disturbances in a population study of type 2 diabetic patients. Eur J Endocrinol.

[B27] Ahuja HS, Szanto A, Nagy L, Davies PJ (2003). The retinoid × receptor and its ligands: versatile regulators of metabolic function, cell differentiation and cell death. J Biol Regul Homeost Agents.

