# Supplementary material for: Ranking candidate genes in rat models of type 2 diabetes
Source: Theor Biol Med Model. 2009 Jul 3;6:12. doi: 10.1186/1742-4682-6-12 (PMC2709893; doi:10.1186/1742-4682-6-12)
Supplement: Additional file 1 — References and keywords. [file 1742-4682-6-12-S1.doc]

**Additional file 1**

**The 24 reference terms used:**

diabetes

diabetic

diabetic foot

diabetic neuropathy

diabetic retinapathy

diabeticnephropathy

glucose transport

glucose uptake

hyperinsulinaemia

hyperinsulinemia

hypoinsulinaemia

hypoinsulinemia

insulin receptor

insulin resistance

insulin sensitivity

Insulinaction

insulinsynthesis

insulin secretion

macroangiopathy

microalbuminuria

microangiopathy

pancreas development

pancreasdifferentiation

proteinuria

The 330 keywords with at a keyword score of at least 0.1 when compared to at least one of the reference terms:

acanthosis nigricans

acarbose

Acetone Bodies

Acidosis Diabetic

Adipocytokines

adiponectin

Adult Progeria

Adult-Onset Diabetes Mellitus

Advanced Glycation End Products

Advanced Glycosylation End Products

african american

Agrimony

air pollution

alcohol consumption

Alloxan Diabetes

alpha Cell Tumor

alpha isoform

alpha-Cell Tumor

American Indians

amputation

Anterior Ischemic Optic Neuropathy

Anti Obesity Agents

Anti Obesity Drugs

Antigen HLA-DR3

Antiobesity Agents

Antiobesity Drugs

Anti-Obesity Drugs

Apolipoprotein B

Apolipoprotein C-III

Arginine Vasopressin

Asians

Atorvastatin

autoantibodies

Autoimmune Diabetes

autosomal

B15

B8

Bay g 5421

BB Rat

BB Rats

BB Wistar Rats

beta locus

beta-cell

Blood Glucose Self Monitoring

blurry vision

Brattleboro Rats

Brittle Diabetes Mellitus

Bronze Diabetes

carbohydrate metabolism

cardiovascular

Carotid Intima-Media Thickness

caucasian

Central Diabetes Insipidus

Cereal grains

Chemotaxis

cholesterol

Corneal Sensitivity

Cushing's Syndrome

Db

dendritic cells

destruction

Diabetes

Diabetes Alloxan

Diabetes Autoimmune

Diabetes Mellitus Brittle

Diabetes Mellitus Insulin Dependent

Diabetes Mellitus Insulin-Dependent

Diabetes Mellitus Maturity Onset

Diabetes Mellitus Maturity-Onset

Diabetes Mellitus Non Insulin Dependent

Diabetes Mellitus Non-Insulin-Dependent

Diabetes Mellitus Type 1

Diabetes Mellitus Type 2

Diabetes Renal

Diabetes Streptozocin

Diabetes Streptozotocin

Diabetic Acidosis

Diabetic Amyotrophy

Diabetic Angiopathy

Diabetic Autonomic Neuropathies

Diabetic Autonomic Neuropathy

Diabetic Diets

Diabetic Feet

Diabetic Foot Ulcer

Diabetic Foot Ulcers

Diabetic Glomerulosclerosis

Diabetic Ketoacidoses

Diabetic Ketosis

Diabetic Microangiopathies

Diabetic Microangiopathy

Diabetic Mononeuropathy

Diabetic Nephropathy

Diabetic Neuralgia

Diabetic Neuropathy

Diabetic Polyneuropathies

Diabetic Polyneuropathy

Diabetic Retinopathies

Diabinese

Diamidine

DIDMOAD

Diet Diabetic

Dietary conjugated linoleic acid

Dietary fat

Dihydrochlorothiazide

Diphenylthiocarbazone

diuresis

DR3

DR4

dry mouth

duct cells

European

Euthyroid Sick Syndrome

Experimental Diabetes Mellitus

fatigue

fatty rats

Foot Diabetic

GAD

Gastric Stasis

gestational

Gestational Diabetes

Gestational Diabetes Mellitus

GK rat

Glucagonomas

Glucophage

glucose

glucose homeostasis

glucose tolerance

glutamic acid decarboxylase

Goto-Kakizaki

haemodialysis

haemodynamic instability

HCTZ

heart rate variability

hepatocyte nuclear factor

high blood pressure

high blood sugar

high cholesterol

Hispanics

HLA DR alpha

HLA DR Antigens

HLA DR beta

HLA DR3 Antigen

HLA DR4 Antigen

HLA-DR

HLA-DR alpha

HLA-DR beta

HLA-DR3

HLA-DR4

HNF3

HNF6

Home Blood Glucose Monitoring

Hydramnios

hyperglycemia

hyperplasia

Hypophosphatemic Rickets

Hypothiazide

IDDM

in Diabetes Obesity

Inbred BB Rats

indian

inflammatory

Infusion Pump

insulin

insulin antibodies

Insulin Resistance Syndrome X

insulin sensitive tissues

Insulin-Dependent Diabetes Mellitus

interleukin-6 receptor

ion channels

IRAP

irbesartan

Iron stores

Ischemic Optic Neuropathy

islet cell

Juvenile-Onset Diabetes Mellitus

kaiser

ketoacidosis

Ketoacidosis Diabetic

ketosis

Ketosis-Prone Diabetes Mellitus

Kidd

Lactic Acidosis

legumes

Leptin signaling

lipid peroxidation

lipoatrophic

Lipoatrophic Diabetes Mellitus

lipoproteins

Lomidine

Long Evans Rats

Long-Evans Rats

low blood sugar

Low T3 Syndrome

Lysine Vasopressin

mannose-binding lectin

Maturity Onset Diabetes Mellitus

Maturity-Onset Diabetes Mellitus

meiosis

Metabolic Cardiovascular Syndrome

metabolic syndrome

Metabolic X Syndrome

metformin

mexican

Mice NOD

Mice Nonobese Diabetic

microalbuminuria

microalbuminuria screening

Microangiopathy Diabetic

MODY

Mortality

Mouse NOD

Mucormycoses

narcolepsy

Necrotizing Renal Papillitis

Nephrogenic Diabetes Insipidus

Nephropathy Diabetic

NeuroD1

Neurogenic Diabetes Insipidus

Neurohypophyseal Diabetes Insipidus

Neuropathy Diabetic

Ngn3

NIDDM

Nkx2,2

Nkx6,1

NOD Mice

NOD Mouse

Non Obese Diabetic Mice

Non Obese Diabetic Mouse

Non-Insulin-Dependent Diabetes Mellitus

Nonobese Diabetic Mice

Non-Obese Diabetic Mice

Nonobese Diabetic Mouse

Non-Obese Diabetic Mouse

Ob

obesity

Octreotide Acetate

Oculomotor Nerve Palsies

Oculomotor Nerve Palsy

Oculomotor Nerve Paralysis

Oculomotor Neuropathy

oligonucleotides

omega-3 fatty acid

open-angle glaucoma

Optic Nerve Ischemia

osmotic

Otsuka Long Evans Tokushima Fatty Rats

Otsuka-Long-Evans-Tokushima Fatty Rats

overweight

oxidation

Painful Diabetic Neuropathies

Painful Diabetic Neuropathy

Pancreas development

Pancreas differentiation

pancreatic

Pax4

Pdx1

Pentamidine Isethionate

Pentamidine Mesylate

Perfusion Pump

Peripheral arterial disease

peroxidation

Phosphate Diabetes

pioglitazone

Pitressin

Pituitary Diabetes Insipidus

Plasma AA

Plasma DHA

Polyneuropathies Diabetic

polyphagia

polyuria

Posterior Ischemic Optic Neuropathy

Postpartum thyroiditis

pregnancy

Pregnancy-Induced Diabetes

prosthetic joint infection

protein kinase C (PKC)

Pump Infusion

Ragaglitazar

Rat BB

Rats BB

Rats Long Evans

Rats Otsuka-Long-Evans-Tokushima

Rats Otsuka-Long-Evans-Tokushima Fatty

renal

Renal Diabetes

Renal Glycosuria

resting energy expenditure

Restless Legs

Retinal Vein Occlusions

Retinal Vein Thrombosis

Retinopathy Diabetic

rosiglitazone

Sandostatin

sawicki

secretion

Self Monitoring Blood Glucose

Self-Monitoring Blood Glucose

Serum gamma-Glutamyltransferase

Sexual dysfunction

SMS 201 995

SMS 201-995

SOD2

somatic growth

Somatostatinomas

Stable Diabetes Mellitus

Statin

Streptozocin Diabetes

Streptozotocin

Streptozotocin Diabetes

Streptozotocine

stroke

superoxide dismutase

Syndrome X Reaven

Tannate

T-cell

Third Nerve Palsy

Third-Nerve Palsies

Third-Nerve Palsy

triglycerides

Troglitazone

ulcer

Vascular

weight loss

Weight Loss Agents

Weight-Loss Agents

Werner's Syndrome

visceral adiposity

Vitamin D-Resistant Rickets

wolfram syndrome

Zucker

The 28 keywords used in the “quick version” of CGC:

acanthosis nigricans

acarbose

adipocytokines

adiponectin

beta-cell

cardiovascular

cholesterol

diabetes

DIDMOAD

gestational

glitazone

glucose

IDDM

insulin

IRAP

ketoacidosis

lipoatrophic

metformin

microalbuminuria

MODY

NeuroD1

NIDDM

obesity

pancreatic

renal

statin

Streptozotocin

vascular
