# Supplementary material for: Ranking candidate genes in rat models of type 2 diabetes
Source: Theor Biol Med Model. 2009 Jul 3;6:12. doi: 10.1186/1742-4682-6-12 (PMC2709893; doi:10.1186/1742-4682-6-12)
Supplement: Additional file 2 — Detailed description of high-ranked genes within the four investigated QTLs. [file 1742-4682-6-12-S2.doc]

### Additional file 2.

**Detailed description of high-ranked genes within the four investigated QTLs.**

### Niddm8

SHC1 *(SHC transforming protein 1) CGC points 195, CGC ranking 1, manual rating 2.* SHC1 was ranked first by the CGC application on the basis of the keywords ”diabetes”, ”glucose tolerance”, ”secretion”, ”insulin”, ”glucose” and ”T-cell”.

It has been shown in Chinese hamster ovary cells that on insulin stimulation the 52-kDa isoform of SHC1 undergoes tyrosin phosphorylation [1].

ENSA *(Endosulfine, alpha) CGC points 129.4, CGC ranking 2, manual rating 2.* ENSA was ranked second by the CGC application on the basis of the keywords ”diabetes” and ”insulin”. ENSA has been proposed to be an endogenous regulator of the K(ATP) channel, which has a key role in the control of insulin release [2].

**Niddm18**

GCK *(Glucokinase)* *CGC points 816.1, CGC ranking 1, manual rating 1.* GCK was ranked first by the CGC application on the basis of the keywords ”diabetes”, ”MODY”, ”gestational”, ”pancreatic”, ”IDDM”, ”NIDDM”, ”caucasian”, ”beta-cell”, ”hyperglycemia”, ”glucose tolerance”, ”glucose homeostasis”, ”secretion”, ”insulin”, ”autosomal”, ”glucose”, ”Insulin-Dependent Diabetes Mellitus” and ”Gestational Diabetes”. GCK transforms glucose to glucose-6-phosphate at the entrance in the cell [3].

## GC (group-specific component) CGC points 189.8, CGC ranking 2, manual rating 1. GC was ranked second by the CGC application on the basis of the keywords ”diabetes”, ”glucose tolerance”, ”insulin” and ”glucose”. GC binds vitamin D which in its metabolically active form is involved in the regulation of insulin levels [4].

*CCKAR (cholecystokinin A receptor) CGC points 168, CGC ranking 3, manual rating 1.*  CCKAR was ranked third by the CGC application on the basis of the keywords ”diabetes”, ”pancreatic”, ”cholesterol”, ”insulin” and ”obesity”. CCKAR is a major physiologic mediator of gall bladder contraction and pancreatic enzyme secretion. A CCKAR mutation has been observed in an African-American patient with obesity and noninsulin-dependent diabetes mellitus [5]. CCKAR is defect in expression in pancreas of OLETF rats [6].

*WFS1 (Wolframin) CGC points 163.3, CGC ranking 4, manual rating 2.* WFS1 was ranked fourth by the CGC application on the basis of the keywords ”diabetes”, ”wolfram syndrome” and ”autosomal”. WFS1 protein may play an important role in both stimulus-secretion coupling for insulin exocytosis as well as maintenance of beta-cell mass [7].

*NKX6A (NK homeobox, family 6, member A) CGC points 123.8, CGC ranking 5, manual rating 1.* NKX6A was ranked fifth by the CGC application on the basis of the keywords ”pancreatic”, ”beta-cell”, ”Nkx6.1”, ”insulin” and ”glucose”.

The rat homolog of NKX6A has been found to suppress glucagon expression and control glucose-stimulated insulin secretion in pancreatic beta cells [8].

**Niddm38**

*RRAD (RAS gene associated with diabetes) CGC points 295.6, CGC ranking 1, manual rating 1.* RRAD was ranked first by the CGC application on the basis of the keywords ”diabetes”, ”IDDM”, ”NIDDM” and ”insulin”. There is a potential synergistic interaction between increased expression of RRAD and high-fat diet in the creation of insulin resistance and the altered lipid metabolism present in type 2 diabetes [9].

*FANCA (Fanconi anemia complementation group A gene) CGC points 175.8, CGC ranking 2, manual rating 2.* FANCA was ranked second by the CGC application on the basis of the keywords ”diabetes”, ”glucose tolerance”, ”renal”, ”secretion”, ”pregnancy”, ”European”, ”autosomal” and ”glucose”. FANCA is part of a nuclear multiprotein core complex which triggers activating monoubiquitination of the FANCD2 protein during the S phase of the growth cycle and after exposure to DNA crosslinking agents. The FA/BRCA pathway is involved in the repair of DNA damage [10]. In a study of 54 patients with Fanconis anemia, 72% had hyperinsulinemia and 25% had impaired glucose tolerance or overt diabetes mellitus [11].

*CETP* (Cholesteryl ester transfer protein, plasma*) CGC points 149.5, CGC ranking 3, manual rating 2.* CETP was ranked third by the CGC application on the basis of the keywords ”diabetes”, ”cardiovascular”, ”Ob”, ”cholesterol”, ”lipoproteins”, ”vascular”, ”renal” and ”Statin”. CETP enables the transfer of cholesteryl esters from HDL to lipoproteins. Plasma CETP mass is associated with plasma leptin in type 2 diabetes [12].

*FOXC2 (forkhead box C2) CGC points 131.5, CGC ranking 4, manual rating 1.* FOXC2 was ranked fourth by the CGC application on the basis of the keywords ”diabetes”, ”insulin” and ”autosomal”. FOXC2 acts as a key regulator of adipocyte metabolism. Increased FOXC2 levels induced by high fat diet seem to counteract most of the symptoms associated with obesity, including hypertriglyceridemia and diet-induced insulin resistance, and a likely consequence would be protection against type 2 diabetes [13].

*HP (*haptoglobin*) CGC points 115.6 CGC ranking 5, manual rating 2.* HP was ranked fifth by the CGC application on the basis of the keywords ”diabetes”, ”cardiovascular”, ”vascular ” and ”oxidation”. HP plays an important role in diabetic vascular disease by binding hemoglobin (Hb) to form a stable Hp-Hb complex and thereby prevent Hb-induced oxidative tissue damage [14]. Recent studies suggest that HP may also serve as a risk factor for type 2 diabetes in certain human populations [15].

**Niddm46**

*GAD1 (Glutamate decarboxylase 1) CGC points 364.9, CGC ranking 1, manual rating 2.* GAD1 was ranked first by the CGC application on the basis of the keywords ”diabetes”, ”NOD Mice”, ”autoantibodies”, ”beta-cell”, ”GAD” and ”autoimmune diabetes”. Beta cell-specific GAD1 expression is required for the development of autoimmune diabetes in NOD mice [16]. It has been suggested that GAD1 is the initiating autoantigen in human type I diabetes because GAD-specific autoantibodies are among the first to appear in the prediabetic phase in human patients [17].

*NEUROD1 (Neurogenic Differentiation 1) CGC points 272.7, CGC ranking 2, manual rating 1.* NEUROD1 was ranked second by the CGC application on the basis of the keywords ”diabetes”, ”MODY”, ”pancreatic”, ”NEUROD1” and ”insulin”.

Following its heterodimerization with the ubiquitous helix-loop-helix HLH protein E47, NEUROD1 regulates insulin gene expression by binding to a critical E-box motif on the insulin promoter [18]. Two heterozygous mutations have been described in NEUROD1 which both were associated with the development of type 2 diabetes [19].

*DPP4 (dipeptidyl peptidase IV) CGC points 221.8, CGC ranking 3, manual rating 1.* DPP4 was ranked third by the CGC application on the basis of the keywords ”diabetes”, ”pancreatic”, ”glucose tolerance”, ”insulin”, ”obesity”, ”glucose” and ”T-cell”. DPP4 may play a critical role in physiologic glucose homeostasis, establishing it as a potential target for therapy in type 2 diabetes [20]. DPP4 inhibition is a viable therapeutic option for the treatment of metabolic disorders related to diabetes and obesity [21].

*MAPK8IP1 (mitogen-activated protein kinase 8-interactin protein) CGC points 193.6, CGC ranking 4, manual rating 1.* MAPK8IP1 was ranked fourth by the CGC application on the basis of the keywords ”pancreatic”, “beta-cell”, “secretion”, “insulin” and “diabetes”. MAPK8IP1 is a key regulator of beta-cell function [22]. In a family with type 2 diabetes individuals in 4 successive generations displayed a change at codon 59 in exon 2 of the MAPK8IP1 gene from AGC (ser) to AAC (asn) [22].

*GCG (Glucagon) CGC points 159.4, CGC ranking 5, manual rating 1.* GCG was ranked fifth by the CGC application on the basis of the keywords ”pancreatic”, ”mortality”, ”insulin”, ”glucose” and ”diabetes”. Glucagon is a pancreatic hormone that counteracts the glucose-lowering action of insulin by stimulating glycogenolysis and gluconeogenesis.

*GPD2 (Glycerol-3-Phosphate Dehydrogenase 2) CGC points 140.9, CGC ranking 6, manual rating 1.* GPD2 was ranked sixth by the CGC application on the basis of the keywords ”pancreatic”, ”insulin”, ”oxidation” and ”diabetes”. GPD2 represents a key component of the pancreatic ß-cell glucose-sensing device. Deficiency of this enzyme appears to contribute to the impairment of glucose-stimulated insulin release in several animal models of noninsulin-dependent diabetes mellitus [23, 24]. A mutation in the GDH2 gene in a patient with type 2 diabetes and his glucose-intolerant half sister has been reported [25].

*CD59 (CD59 antigen P18-20) CGC points 121.9, CGC ranking 7, manual rating 3.* CD59 was ranked seventh by the CGC application on the basis of the keywords ”vascular”, ”glucose”, ”diabetes” and ”T-cell”. Insertion of the MAC (Membrane Attack Complex) into endothelial cell membranes causes the release of growth factors that stimulate tissue growth and proliferation. The complement regulatory membrane protein CD59 restricts MAC formation. Increased glucose levels in diabetes patients cause protein glycation, which may inhibit CD59. If so, glycation-inactivation of CD59 would cause increased MAC deposition and MAC-stimulated cell proliferation. This may help explain the distinct propensity of humans to develop vascular proliferative complications of diabetes [26].

*CAT (Catalase) CGC points 109.3, CGC ranking 8, manual rating 2.* CAT was ranked eighth by the CGC application on the basis of the keywords ”pancreatic” and ”diabetes”. An increased frequency of diabetes in catalase-deficient Hungarian patients as compared to unaffected first-degree relatives and the general Hungarian population has been reported. Quantitative deficiency of catalase might predispose to cumulative oxidant damage of pancreatic beta cells and resulting diabetes [27].

*FUT7 (Fucosyltransferase 7) CGC points 105.6, CGC ranking 9, manual rating 3.* FUT7 was ranked ninth by the CGC application on the basis of the keywords ”vascular”, ”diabetes” and ”ulcer”. FUT7 synthesize the sialyl Lewis x oligosaccharide determinant that is an essential component for mediating adhesion of leukocytes. A homozygous recessive individual was found to have ulcer disease, noninsulin-dependent diabetes, osteoporosis, spondyloarthrosis, and Sjogren syndrome, but the relationship between disease and the mutation is not clear [28].

1. Migliaccio E, Mele S, Salcini AE, Pelicci G, Lai KM, Superti-Furga G, Pawson T, Di Fiore PP, Lanfrancone L, Pelicci PG: **Opposite effects of the p52shc/p46shc and p66shc splicing isoforms on the EGF receptor-MAP kinase-fos signalling pathway**. *Embo J* 1997, **16**(4):706-716.

2. Heron L, Virsolvy A, Peyrollier K, Gribble FM, Le Cam A, Ashcroft FM, Bataille D: **Human alpha-endosulfine, a possible regulator of sulfonylurea-sensitive KATP channel: molecular cloning, expression and biological properties**. *Proc Natl Acad Sci U S A* 1998, **95**(14):8387-8391.

3. Matschinsky FM: **Glucokinase as glucose sensor and metabolic signal generator in pancreatic beta-cells and hepatocytes**. *Diabetes* 1990, **39**(6):647-652.

4. Szathmary EJ: **The effect of Gc genotype on fasting insulin level in Dogrib Indians**. *Hum Genet* 1987, **75**(4):368-372.

5. Inoue H, Iannotti CA, Welling CM, Veile R, Donis-Keller H, Permutt MA: **Human cholecystokinin type A receptor gene: cytogenetic localization, physical mapping, and identification of two missense variants in patients with obesity and non-insulin-dependent diabetes mellitus (NIDDM)**. *Genomics* 1997, **42**(2):331-335.

6. Funakoshi A, Miyasaka K, Shinozaki H, Masuda M, Kawanami T, Takata Y, Kono A: **An animal model of congenital defect of gene expression of cholecystokinin (CCK)-A receptor**. *Biochem Biophys Res Commun* 1995, **210**(3):787-796.

7. Ishihara H, Takeda S, Tamura A, Takahashi R, Yamaguchi S, Takei D, Yamada T, Inoue H, Soga H, Katagiri H *et al*: **Disruption of the WFS1 gene in mice causes progressive beta-cell loss and impaired stimulus-secretion coupling in insulin secretion**. *Hum Mol Genet* 2004, **13**(11):1159-1170. Epub 2004 Mar 1131.

8. Schisler JC, Jensen PB, Taylor DG, Becker TC, Knop FK, Takekawa S, German M, Weir GC, Lu D, Mirmira RG *et al*: **The Nkx6.1 homeodomain transcription factor suppresses glucagon expression and regulates glucose-stimulated insulin secretion in islet beta cells**. *Proc Natl Acad Sci U S A* 2005, **102**(20):7297-7302. Epub 2005 May 7299.

9. Ilany J, Bilan PJ, Kapur S, Caldwell JS, Patti ME, Marette A, Kahn CR: **Overexpression of Rad in muscle worsens diet-induced insulin resistance and glucose intolerance and lowers plasma triglyceride level**. *Proc Natl Acad Sci U S A* 2006, **103**(12):4481-4486. Epub 2006 Mar 4414.

10. Folias A, Matkovic M, Bruun D, Reid S, Hejna J, Grompe M, D'Andrea A, Moses R: **BRCA1 interacts directly with the Fanconi anemia protein FANCA**. *Hum Mol Genet* 2002, **11**(21):2591-2597.

11. Wajnrajch MP, Gertner JM, Huma Z, Popovic J, Lin K, Verlander PC, Batish SD, Giampietro PF, Davis JG, New MI *et al*: **Evaluation of growth and hormonal status in patients referred to the International Fanconi Anemia Registry**. *Pediatrics* 2001, **107**(4):744-754.

12. Dullaart RP, de Vries R, Dallinga-Thie GM, van Tol A, Sluiter WJ: **Plasma cholesteryl ester transfer protein mass and phospholipid transfer protein activity are associated with leptin in type 2 diabetes mellitus**. *Biochim Biophys Acta* 2007, **1771**(1):113-118.

13. Cederberg A, Gronning LM, Ahren B, Tasken K, Carlsson P, Enerback S: **FOXC2 is a winged helix gene that counteracts obesity, hypertriglyceridemia, and diet-induced insulin resistance**. *Cell* 2001, **106**(5):563-573.

14. Asleh R, Marsh S, Shilkrut M, Binah O, Guetta J, Lejbkowicz F, Enav B, Shehadeh N, Kanter Y, Lache O *et al*: **Genetically determined heterogeneity in hemoglobin scavenging and susceptibility to diabetic cardiovascular disease**. *Circ Res* 2003, **92**(11):1193-1200. Epub 2003 May 1115.

15. Quaye IK, Ababio G, Amoah AG: **Haptoglobin 2-2 phenotype is a risk factor for type 2 diabetes in Ghana**. *J Atheroscler Thromb* 2006, **13**(2):90-94.

16. Yoon JW, Yoon CS, Lim HW, Huang QQ, Kang Y, Pyun KH, Hirasawa K, Sherwin RS, Jun HS: **Control of autoimmune diabetes in NOD mice by GAD expression or suppression in beta cells**. *Science* 1999, **284**(5417):1183-1187.

17. von Boehmer H, Sarukhan A: **GAD, a single autoantigen for diabetes**. *Science* 1999, **284**(5417):1135, 1137.

18. Naya FJ, Stellrecht CM, Tsai MJ: **Tissue-specific regulation of the insulin gene by a novel basic helix-loop-helix transcription factor**. *Genes Dev* 1995, **9**(8):1009-1019.

19. Malecki MT, Jhala US, Antonellis A, Fields L, Doria A, Orban T, Saad M, Warram JH, Montminy M, Krolewski AS: **Mutations in NEUROD1 are associated with the development of type 2 diabetes mellitus**. *Nat Genet* 1999, **23**(3):323-328.

20. Marguet D, Baggio L, Kobayashi T, Bernard AM, Pierres M, Nielsen PF, Ribel U, Watanabe T, Drucker DJ, Wagtmann N: **Enhanced insulin secretion and improved glucose tolerance in mice lacking CD26**. *Proc Natl Acad Sci U S A* 2000, **97**(12):6874-6879.

21. Conarello SL, Li Z, Ronan J, Roy RS, Zhu L, Jiang G, Liu F, Woods J, Zycband E, Moller DE *et al*: **Mice lacking dipeptidyl peptidase IV are protected against obesity and insulin resistance**. *Proc Natl Acad Sci U S A* 2003, **100**(11):6825-6830. Epub 2003 May 6814.

22. Waeber G, Delplanque J, Bonny C, Mooser V, Steinmann M, Widmann C, Maillard A, Miklossy J, Dina C, Hani EH *et al*: **The gene MAPK8IP1, encoding islet-brain-1, is a candidate for type 2 diabetes**. *Nat Genet* 2000, **24**(3):291-295.

23. Malaisse WJ: **Is type 2 diabetes due to a deficiency of FAD-linked glycerophosphate dehydrogenase in pancreatic islets?** *Acta Diabetol* 1993, **30**(1):1-5.

24. Malaisse WJ: **Non-insulin-dependent diabetes mellitus and islet B-cell mitochondrial glycerophosphate dehydrogenase deficiency**. *Diabet Med* 1995?, **12**(6):479-481.

25. Novials A, Vidal J, Franco C, Ribera F, Sener A, Malaisse WJ, Gomis R: **Mutation in the calcium-binding domain of the mitochondrial glycerophosphate dehydrogenase gene in a family of diabetic subjects**. *Biochem Biophys Res Commun* 1997, **231**(3):570-572.

26. Acosta J, Hettinga J, Fluckiger R, Krumrei N, Goldfine A, Angarita L, Halperin J: **Molecular basis for a link between complement and the vascular complications of diabetes**. *Proc Natl Acad Sci U S A* 2000, **97**(10):5450-5455.

27. Goth L, Eaton JW: **Hereditary catalase deficiencies and increased risk of diabetes**. *Lancet* 2000, **356**(9244):1820-1821.

28. Bengtson P, Larson C, Lundblad A, Larson G, Pahlsson P: **Identification of a missense mutation (G329A;Arg(110)--> GLN) in the human FUT7 gene**. *J Biol Chem* 2001, **276**(34):31575-31582.
